# Doping of Sn-based two-dimensional perovskite semiconductor for high-performance field-effect transistors and thermoelectric devices

**DOI:** 10.1016/j.isci.2022.104109

**Published:** 2022-03-17

**Authors:** Yu Liu, Ping-An Chen, Xincan Qiu, Jing Guo, Jiangnan Xia, Huan Wei, Haihong Xie, Shijin Hou, Mai He, Xiao Wang, Zebing Zeng, Lang Jiang, Lei Liao, Yuanyuan Hu

**Affiliations:** 1 Key Laboratory for Micro/Nano Optoelectronic Devices of Ministry of Education & International Science and Technology Innovation Cooperation Base for Advanced Display Technologies of Hunan Province, School of Physics and Electronics, Hunan University, Changsha 410082, China; 2 Shenzhen Research Institute of Hunan University, Shenzhen 518063, China; 3 State Key Laboratory of Chemo/Biosensing and Chemometrics, College of Chemistry and Chemical Engineering, Hunan University, Changsha 410082, China; 4 Beijing National Laboratory for Molecular Sciences, Key Laboratory of Organic Solids, Institute of Chemistry, Chinese Academy of Sciences, Beijing 100190, China

**Keywords:** Inorganic materials, Materials science, Materials chemistry, Devices

## Abstract

Doping is an important technique for semiconductor materials and devices, yet effective and controllable doping of organic-inorganic halide perovskites is still a challenge. Here, we demonstrate a facile way to dope two-dimensional Sn-based perovskite (PEA)_2_SnI_4_ by incorporating SnI_4_ in the precursor solutions. It is observed that Sn^4+^ produces p-doping effect on the perovskite, which increases the electrical conductivity by 10^5^ times. The dopant SnI_4_ is also found to improve the film morphology of (PEA)_2_SnI_4_, leading to reduced trap states. This doping technique allows us to improve the room temperature mobility of (PEA)_2_SnI_4_ field-effect transistors from 0.25 to 0.68 cm^2^ V^−1^ s^−1^ thanks to reduced trapping effects in the doped devices. Moreover, the doping technique enables the characterization and improvement of the thermoelectric performance of (PEA)_2_SnI_4_ films, which show a high power factor of 3.92 μW m^−1^ K^−2^ at doping ratio of 5 mol %.

## Highlights

• The p-doping effect of Sn^4+^ on (PEA)_2_SnI_4_ is demonstrated and confirmed.
• Proper Sn^4+^ doping can improve the mobilities of (PEA)_2_SnI_4_ FETs.
• Thermoelectric performance of (PEA)_2_SnI_4_ is characterized and enhanced by Sn^4+^ doping.

## Graphical abstract

**Figure:**
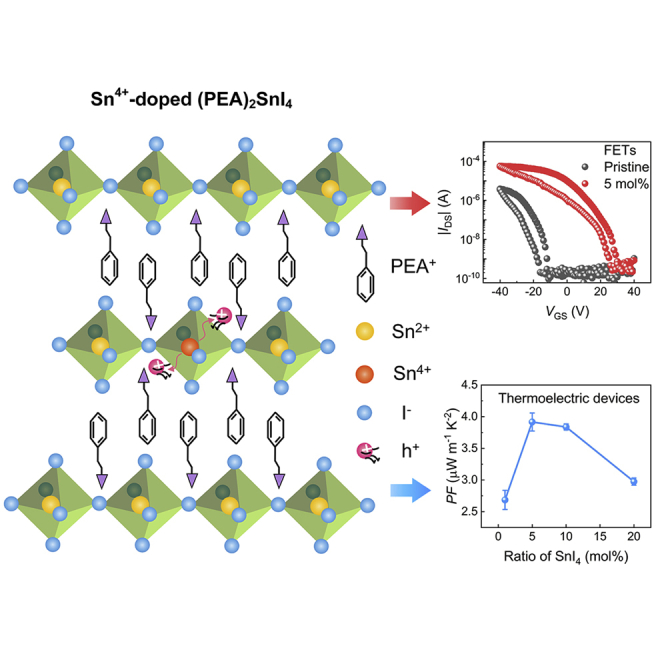

## Introduction

Organic-inorganic hybrid perovskite (OIHP) semiconductors have been extensively studied in the field of solar cells, light-emitting diodes, and photodetectors due to their excellent optoelectronic properties, such as large light-absorption coefficients, weakly bound excitons, long charge-carrier diffusion length, and tunable bandgap (García de Arquer et al., 2017; Liu et al., 2020; Ni et al., 2020; Wang et al., 2019b; Zhao et al., 2020). The low effective mass and intrinsically high carrier mobility of perovskite semiconductors also make them especially attractive as the semiconductor layers in field-effect transistors (FETs) (Senanayak et al., 2020; She et al., 2020; Wang et al., 2019a).

Among the various perovskite semiconductors, Sn-based two-dimensional (2D) layered perovskites have been the subject of interest for their promising applications in high-mobility FETs (Kagan et al., 1999; Matsushima et al., 2016; Zhu et al., 2020b). Compared to their three-dimensional (3D) analogs, 2D perovskite possesses good environmental stability because the bulky hydrophobic organic cationic chains between inorganic octahedral cages can prevent moisture and oxygen from intruding into the materials (Liu et al., 2021). Meanwhile, the ion migration causing the gate-field screening effect, which is believed to be a bottleneck for 3D perovskite FETs (Senanayak et al., 2017; Zeidell et al., 2018), is minimized in 2D perovskite since bulky organic chains can effectively inhibit the ion migration along the out-of-plane direction (Lin et al., 2017; Matsushima et al., 2016; Tsai et al., 2016). More interestingly, the organic ligands in 2D perovskites can be structurally engineered, which provides a paradigm for tuning the properties of perovskites through structure engineering (Gao and Dou, 2021; Gao et al., 2019a, 2019b; Liang et al., 2021). For example, Dou et al. demonstrated that the introduction of bulky conjugated organic ligands can enhance the stability as well as the carrier mobility of Sn-based 2D perovskites relative to short aliphatic chains (Gao et al., 2019b; Liang et al., 2021). As the most heavily studied Sn-based 2D perovskite, (C_6_H_5_C_2_H_4_NH_3_)_2_SnI_4_ ((PEA)_2_SnI_4_) has attracted a lot of interest since the first report of (PEA)_2_SnI_4_ FETs with mobility of 0.6 cm^2^ V^−1^ s^−1^ by Kagan et al., in 1999 (Kagan et al., 1999), and hole mobility as high as 15 cm^2^ V^−1^ s^−1^ was reported in carefully designed (PEA)_2_SnI_4_ FETs (Matsushima et al., 2016), indicating the great potential of this semiconductor for flexible, printable, large-area and low-cost thin-film transistors.

As well known, doping is an essential technique for semiconductors and devices since it can provide efficient adjustment to the electrical properties of semiconductors in terms of carrier concentration and mobility. As a typical example of doping, silicon doping takes the form of atomic substitution by thermal diffusion or ion implantation and now is an indispensable technique in the modern microelectronics industry. Doping has also been intensively studied in organic semiconductor counterparts due to the great potential benefits that can be brought by the technique (Guo et al., 2021; Jacobs and Moule, 2017; Sakai et al., 2021; Wei et al., 2021; Yamashita et al., 2019). Being an emerging semiconductor, (PEA)_2_SnI_4_ provides new opportunities and platforms for studying doping physics and techniques, which will not only help enhance the performance of (PEA)_2_SnI_4_ FETs but also expand its applications in other optoelectronic devices.

Previously, Qin et al. reported the substitutional doping of (PEA)_2_SnI_4_ by Pb^2+^, which leads to improved environmental stability of (PEA)_2_SnI_4_ FETs but depressed hole transport owing to the larger effective mass of Pb and higher contact resistance in the devices (Qin et al., 2021). Reo et al. considered doping of (PEA)_2_SnI_4_ thin films with Cu^+^ by adding copper iodide (CuI) in the precursor solution (Reo et al., 2021). It turns out that the hole transport in (PEA)_2_SnI_4_ FETs gets enhanced with the incorporation of CuI. However, further density functional theory (DFT) calculations and X-ray diffraction (XRD) measurements reveal that the Cu^+^ is likely distributed at grain boundaries instead of replacing the Sn^2+^ within the perovskite lattices, and the improved FET performance is more likely attributed to the outstanding hole-transport property of CuI rather than doping (Reo et al., 2021). These studies indicate doping can be a powerful technique to tune or enhance the performance of (PEA)_2_SnI_4_ devices, but presently effective doping strategies for (PEA)_2_SnI_4_ remain to be established.

Herein, we report effective p-doping of (PEA)_2_SnI_4_ 2D layered perovskite with Sn^4+^ through substituting part of the SnI_2_ with SnI_4_ in the precursor solution. The doping effect of Sn^4+^ was confirmed by electrical and spectroscopy characterizations. In addition to the doping effect, the incorporation of SnI_4_ was revealed to improve the film morphology of (PEA)_2_SnI_4_. This doping strategy was then used to fabricate bottom-gate bottom-contact (BGBC) (PEA)_2_SnI_4_ FETs, which leads to an increase of average mobility from 0.25 cm^2^ V^−1^ s^−1^ for pristine devices to 0.68 cm^2^ V^−1^ s^−1^ for devices doped with 5 mol % SnI_4_. The effect of doping on charge injection and charge transport processes in the FET devices are also investigated and clarified. Moreover, the doping technique allows us to characterize the thermoelectric (TE) performance of (PEA)_2_SnI_4_, and Seebeck coefficients varying between 387 and 660 μV K^−1^ were observed, with the maximum power factor reaching 3.92 μW m^−1^ K^−2^, which demonstrates the promising usage of 2D perovskite semiconductors for high-performance TE devices. It is notable that a similar doping strategy was reported in (4Tm)_2_FASn_2_I_7_ for high-performance TE devices during the preparation of this manuscript (Hsu et al., 2021), which together with this study highlights the great potential of the SnI_4_-doping technique to be used in 2D Sn-based perovskite for low-cost and high-performance devices.

## Results and discussion

### Doping of (PEA)_2_SnI_4_ films by SnI_4_

**Figure 1:**
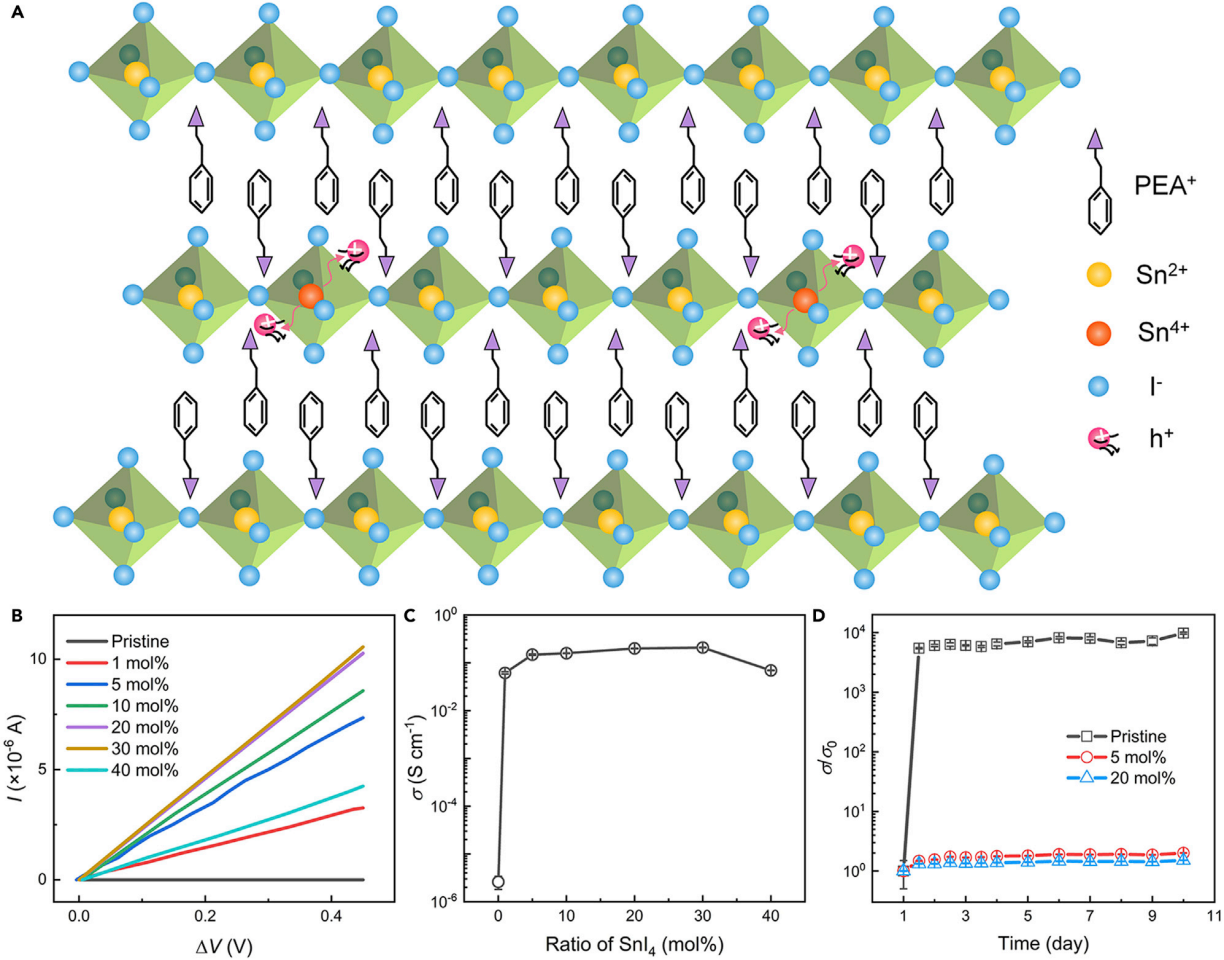
Confirmation of the doping effect of SnI_4_ on (PEA)_2_SnI_4_ (A) Schematic diagram showing the structure of (PEA)_2_SnI_4_ and the SnI_4_ doping mechanism. (B) Current-voltage curves of perovskite films with different SnI_4_ ratios. (C) Electrical conductivities as a function of doping ratio calculated from (B). (D) The relative conductivity changes (*σ*/*σ*_0_) of pristine, 5 mol % and 20 mol % SnI_4_-doped films with time when they were stored in Ar glove box.

Figure 1A illustrates the structure of (PEA)_2_SnI_4_, in which the corner-sharing [SnI_6_]^4−^ octahedra are alternated by a bilayer of PEA^+^ organic cations. Previously, it was reported that Sn^2+^ in Sn-based perovskites are easily oxidized to Sn^4+^, which are metastable and can thermodynamically evolve to the Sn^2+^, releasing two holes in the valence band and leading to the p-doping of the perovskite, *i.e.,* Sn^4+^ → Sn^2+^ + 2h^+^ (Hsu et al., 2021; Ricciarelli et al., 2020; Stoumpos et al., 2013). These results motivate us to intentionally incorporate Sn^4+^ into (PEA)_2_SnI_4_ for controllable p-doping. For such purpose, Sn^4+^ was introduced by mixing phenethylammonium iodide (PEAI), SnI_2_ and SnI_4_ with mol ratio of 2:1−*x*:*x* (0 *< x <* 1) in a mixture of dimethylformamide (DMF) and N-Methyl-2-pyrrolidone (NMP) with volume ratio of 3:1, where *x* represents the doping ratio of SnI_4_.

The measured electrical conductivities (*σ*) of (PEA)_2_SnI_4_ films doped with different doping ratios are shown in Figure 1C (see Figure S1 for more information), with the current-voltage curves shown in Figure 1B. The electrical conductivity of the pristine film is 2.6 × 10^−6^ S cm^−1^, and it is enhanced as the doping ratio increases, reaching a maximum value of 2.1 × 10^−1^ S cm^−1^ at the doping ratio of 30 mol %, which is direct evidence showing the doping effect of SnI_4_ on (PEA)_2_SnI_4_ perovskite. It should be mentioned that extra I^−^ was also introduced into the films together with Sn^4+^, and I^−^ was reported to greatly improve the charge transport property of (PEA)_2_SnI_4_ by compensating the iodine vacancies (V_I_), which behave as trap states (Reo et al., 2021). So, it might be argued that I^−^ may also contribute to the enhancement of conductivity in the doped (PEA)_2_SnI_4_ films. However, the dramatic increase of conductivity about five orders of magnitude in doped films suggests that it should be Sn^4+^ (doping effect) rather than I^−^ (trap passivation effect) that plays a dominant role. We then investigated the stability of the doped (PEA)_2_SnI_4_ films, with the results of relative conductivity changes (*σ*/*σ*_0_) shown in Figure 1D, where *σ*_0_ is the initial conductivity. It is seen that compared to the pristine (PEA)_2_SnI_4_ films, which are prone to be oxidized (Sn^2+^ → Sn^4+^) and experience conductivity increase even though they are stored in an Ar-filled glove box (Zhu et al., 2020b), the doped (PEA)_2_SnI_4_ films show better stability of the conductivity.

### Structural and spectroscopic characterizations on doped (PEA)_2_SnI_4_ films

**Figure 2:**
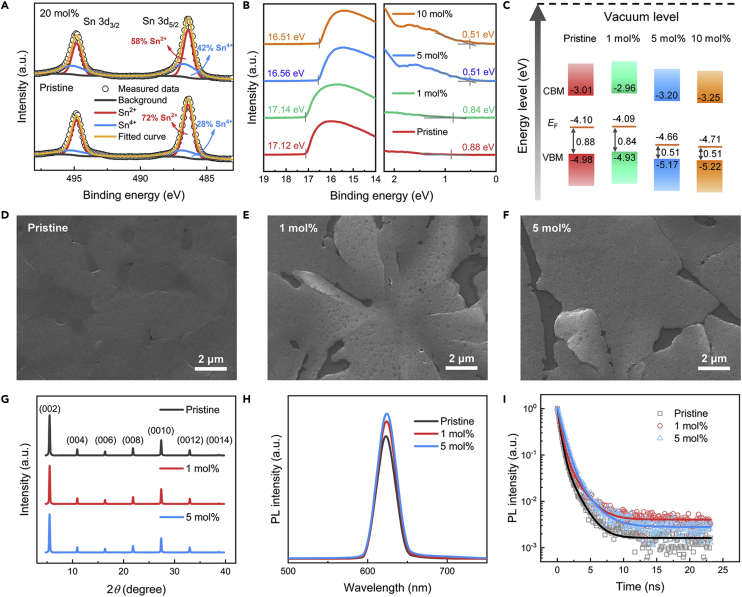
Characterizations of the doping effect of SnI_4_ on (PEA)_2_SnI_4_ (A) XPS results of Sn 3d for pristine and 20 mol % SnI_4_-doped (PEA)_2_SnI_4_ films. The fitting results of the peaks are illustrated in the figure, which show the ratio changes of SnI_4_ upon doping. (B) UPS results of pristine, 1, 5, and 10 mol % SnI_4_-doped perovskite films. (C–F) (C) Energy level structures of pristine, 1, 5, and 10 mol % SnI_4_-doped perovskite films. SEM images of (D) pristine (PEA)_2_SnI_4_ films, (E) 1 mol %, and (F) 5 mol % SnI_4_-doped (PEA)_2_SnI_4_ films (scale bar: 2 μm). (G–I) (G) XRD patterns, (H) steady-state PL, and (I) time-resolved PL of (PEA)_2_SnI_4_ thin films with different doping ratios.

To further understand the doping effect of SnI_4_ on (PEA)_2_SnI_4_ films, X-ray photoelectron spectroscopy (XPS) was used to identify the composition of Sn element in the perovskite films. Figure 2A demonstrates the XPS spectra of Sn 3d for pristine and 20 mol % SnI_4_-doped (PEA)_2_SnI_4_ films. We find that the Sn^4+^ content of the doped (PEA)_2_SnI_4_ films is increased from 28 atomic % (at %) (for pristine ones) to 42 at % (for 20 mol%-doped ones), indicating the incorporation of Sn^4+^ during the film formation. Furthermore, UV photoelectron spectroscopy (UPS) measurements were performed to study the effects of doping on energy levels of perovskite films (Figure 2B). The Fermi level (*E*_F_), valence band maximum (VBM), and conduction band minimum (CBM) for pristine films and films doped with 1 mol % and 5 mol % SnI_4_ are shown in Figure 2C. The optical bandgap (*E*_g_) for all the films were measured to be about 1.97 eV through absorption spectra (see more details in the Figure S2), which is consistent with the previously reported values (Zhang et al., 2021). One important result of UPS measurements is that the Fermi level moves closer to the VBM as the doping ratio increases, indicating the increased concentration of holes upon SnI_4_ doping. We also note that the shifting of Fermi level becomes almost saturated when the doping ratio is higher than 5 mol %, which is in line with the saturation of electrical conductivity above this doping ratio as shown in Figure 1C.

Furthermore, we inspected the influence of SnI_4_ on the morphology and structure of (PEA)_2_SnI_4_ films. From scanning electron microscopy (SEM) images of the perovskite films, it is seen that the domain is enlarged upon the SnI_4_ doping (Figures 2D–2F), which is an indication of fewer domain boundaries and trap states. The corresponding atomic force microscopy (AFM) images also exhibit more uniform surface morphology with reduced roughness as doping ratio increases (Figure S3). To further understand the effect of SnI_4_ on film structure, we used X-ray diffraction (XRD) to probe the film crystallinity. As shown in Figure 2G, XRD patterns of pristine and doped (PEA)_2_SnI_4_ films show similar diffraction peaks, which are assigned to the strong (0 0 *l*) (*l* = 2, 4, 6, 8, 10, 12, 14) diffractions at 5.5°, 10.9°, 16.4°, 21.9°, 27.4°, 33.0°, and 38.7°, respectively, indicating a layered structure of the films (Reo et al., 2021; Zhu et al., 2020a). However, the full width at half maximum (FWHM) of (0 0 2) peaks are found to be 0.197°, 0.183°, and 0.182° for pristine, 1 mol %, and 5 mol % SnI_4_-doped films, respectively. This decreased FWHM implies a larger crystallite size in doped films, which is in accordance with the SEM results.

In addition, we measured the steady-state and time-resolved photoluminescence (PL) spectra of (PEA)_2_SnI_4_ films prepared with and without SnI_4_ dopants. From the steady-state PL spectra (Figure 2H), it is evident that the quenching of the perovskite emission signal is reduced in doped films, suggesting there are fewer traps causing non-radiative recombination. The time-resolved PL data (Figure 2I) are found to fit well to the biexponential decay equation:

(Equation 1) $$I\left(t\right)=A_{1}\;\exp\left(-\frac{t}{\tau_{1}}\right)+A_{2}\;\exp\left(-\frac{t}{\tau_{2}}\right)$$

where *A*_1_ and *A*_2_ are relative amplitudes, *τ*_1_ and *τ*_2_ are carrier lifetimes for fast and slow decay, respectively, and *I*(*t*) is the PL intensity (Bi et al., 2016). The carrier lifetimes of *τ*_1_ (*τ*_2_) for the pristine films and films doped with 1 mol % and 5 mol % SnI_4_ are 0.40 ns (1.46 ns), 0.50 ns (1.74 ns), and 0.72 ns (2.10 ns), respectively. The longer PL lifetime of doped (PEA)_2_SnI_4_ films further demonstrates that the addition of SnI_4_ can reduce the trap density in the films, consistent with the results of steady-state PL. Such reduction of trap density in the SnI_4_-doped films is not unexpected since both the filling of trap states by dopants and improved crystallinity and morphology of the doped films can lower the trap density.

### Doping of (PEA)_2_SnI_4_ films for FETs

**Figure 3:**
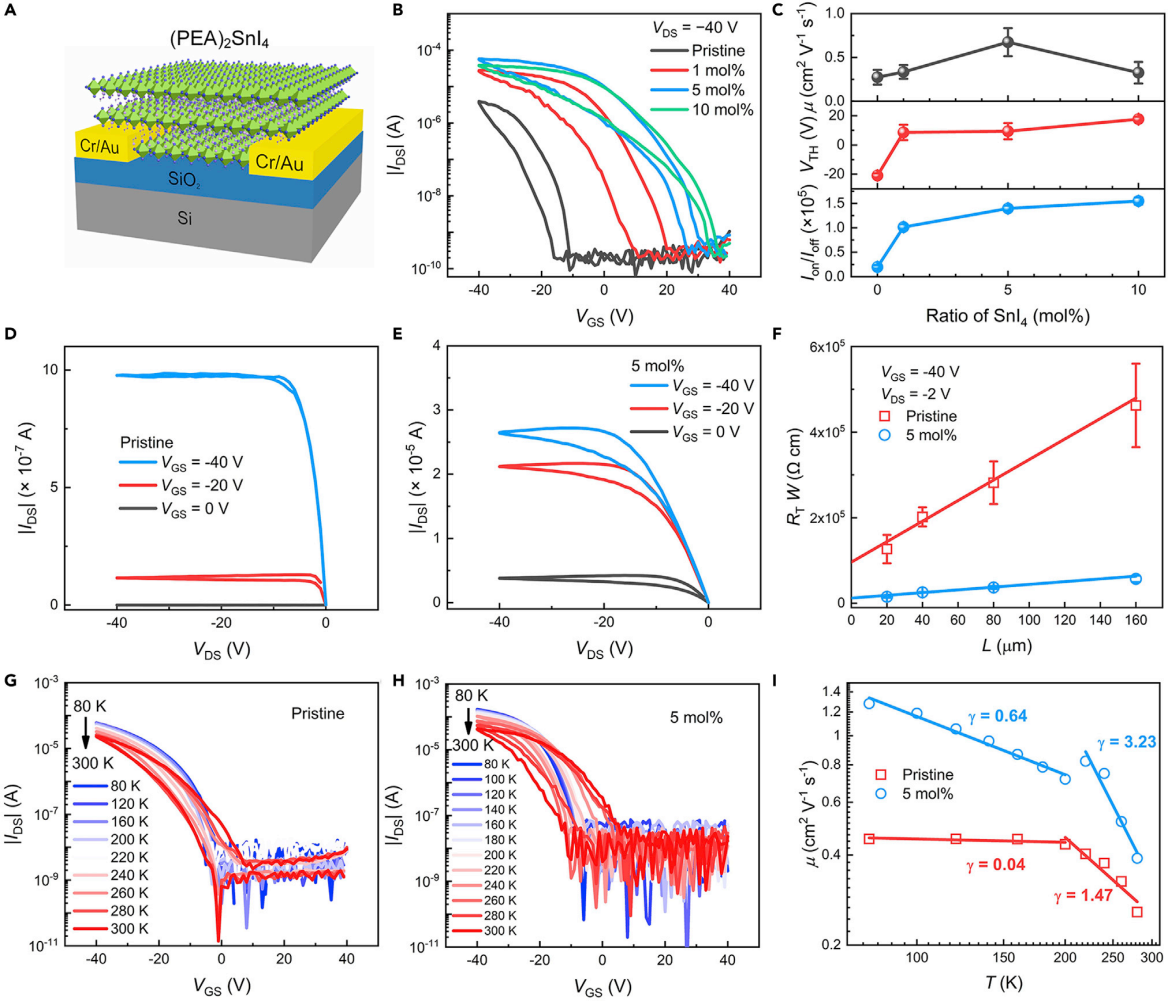
Fabrication and characterization of (PEA)_2_SnI_4_ FETs (A) Device configuration of (PEA)_2_SnI_4_ BGBC FET. (B) Transfer curves (*V*_DS_ = −40 V) of FETs with different doping ratios. (C–E) (C) Mobilities, threshold voltages, and on/off ratios extracted from transfer characteristics. Output curves of the (D) pristine and (E) 5 mol % SnI_4_-doped devices. (F–H) (F) The *L*-dependent total resistance (*R*_T_*W*) of pristine and 5 mol % SnI_4_-doped FETs. Temperature-dependent FETs transfer characteristics of (G) pristine FETs and (H) 5 mol % SnI_4_-doped FETs from 80 K to 300 K. (I) Temperature-dependent mobility extracted from the transfer curves of pristine and 5 mol % SnI_4_-doped FETs.

Encouraged by the prominent doping effect of SnI_4_ on (PEA)_2_SnI_4_ films and the accompanied improvement of film quality, we sought to lever these benefits to enhance the device performance of (PEA)_2_SnI_4_ FETs. Figure 3A shows the BGBC device structure used in this study. Si^++^/SiO_2_ (300 nm) were used as substrates, and photolithography-defined Cr/Au (2 nm/30 nm) were used as source/drain electrodes. Perovskite precursors were spin-coated on the substrates at 4000 rpm for 30 s and then annealed at 100°C for 10 min in a glove box. The devices with pristine (PEA)_2_SnI_4_ films as active layers show typical p-type FET behaviors due to the low formation energy of tin vacancies which can lead to self-doping effect (see Figure 3B) (Euvrard et al., 2021; Takahashi et al., 2011). The transfer plots (square root of current) for mobility calculations are shown in Figure S5A. The saturation mobility (*μ*) of 0.25 ± 0.08 cm^2^ V^−1^ s^−1^, threshold voltage (*V*_TH_) of −21 ± 1.6 V, and on/off ratio (*I*_on_/*I*_off_) of 10^4^ (channel length *L* = 160 μm, channel width *W* = 1000 μm) were obtained in the devices, as shown in Figure 3C. These performance merits are similar to those reported in previous studies (Qin et al., 2021; Zhang et al., 2019; Zhu et al., 2020b).

By doping the films with SnI_4_ in the doping range of 1–5 mol %, we observed a significant enhancement of the device current, indicating the improvement of device performance. However, the current begins to decay when the doping ratio is higher than 10 mol % (Figures 3B and S4). The variation of performance parameters as a function of doping ratio is presented in Figure 3C, which shows the devices exhibit *μ* of 0.68 ± 0.16 cm^2^ V^−1^ s^−1^, *V*_TH_ of 10 ± 5.6 V, and *I*_on_/*I*_off_ of 10^5^ at the optimized doping ratio of 5 mol %. In addition, the doping-ratio-dependent carrier concentration (*p*) (see Figure S6) calculated from the formula *σ* = *pqμ*, where *q* is the unit charge, exhibits a much higher *σ* upon doping with 1 mol % SnI_4_ and a saturation trend with higher doping ratios. The output curves of the pristine and 5 mol % SnI_4_-doped devices are shown in Figures 3D and 3E, respectively, and output curves of 1 mol % and 10 mol % SnI_4_-doped devices are shown in Figures S5B and S5C. Thus, we can see that the currents of output curves increase in FETs with higher doping ratios. One thing notable is the increased hysteresis in the doped device, which is probably attributed to the extra I^−^ brought by SnI_4_ in the films, causing enhanced ion migration. In spite of the slightly increased hysteresis, these results still show that doping (PEA)_2_SnI_4_ films with SnI_4_ is a promising way to achieve high-performance FETs.

Following that, we conducted investigations to deeply understand how the doping affects the performance of (PEA)_2_SnI_4_ FETs. First, we examined the influence of doping on contact resistance (*R*_C_*W*) in the devices through the transmission line method (TLM) (Bhargava and Singh, 2014; Minari et al., 2006; Xu et al., 2010). As shown in Figure 3F, the contact resistance for pristine devices is 9.6 × 10^4^ Ω cm, and it is reduced to 1.2 × 10^4^ Ω cm for the 5 mol % SnI_4_-doped devices. The average total resistance (*R*_T_*W*) of pristine and 5 mol % SnI_4_-doped FETs with *L* = 160 μm are 4.6 × 10^5^ Ω cm and 5.7 × 10^4^ Ω cm, respectively, by which the average channel resistance (*R*_Ch_*W* = *R*_T_*W* – *R*_C_*W*) are obtained to be 3.6 × 10^5^ Ω cm and 4.5 × 10^4^ Ω cm, respectively. Interestingly, the ratio of *R*_C_*W* of FETs before and after doping is about 8.0, which is similar to that of *R*_Ch_*W*, indicating that doping has similar effects on *R*_C_*W* and *R*_Ch_*W*. The reduction of contact resistance and channel resistance are expected when doping of the semiconductor layer occurs in FETs (Hu et al., 2018a, 2018b; Kim et al., 2016, 2019).

Additionally, we inspected the charge transport in the pristine and doped devices by performing temperature-dependent measurements on their electrical characteristics (*L* = 80 μm, *W* = 2000 μm). It should be noted that the transfer curves of pristine and 5 mol % SnI_4_-doped FETs change after vacuuming, as shown in Figure S7. With the temperature increasing from 80 K to 300 K, the pristine device exhibits reduced *I*_on_ (at *V*_G_ = −40 V) and increased hysteresis (see Figure 3G). The cooling process from 300 K to 80 K also shows the same trend (see Figure S8). This increase of hysteresis is possibly attributed to the enhanced ion migration in the high-temperature regime although 2D perovskites have been reported to show inhibited ion migration compared to their 3D analogs (Liu et al., 2021). The extracted mobility and its temperature dependence are presented in Figure 3I, which illustrates an increase of *μ* with decreasing temperature. In general, the temperature dependence of charge-carrier mobility is closely related to the charge scattering mechanism in the semiconductors and can be expressed as $\mu \propto T^{-\gamma }$ (Biewald et al., 2019; Wright et al., 2016), where *γ* reflects the magnitude of charge scattering, with larger *γ* representing stronger scattering in the perovskite (Buizza et al., 2021; Herz, 2017; Senanayak et al., 2017). The pristine device shows a *γ* value of 1.47 between 200 and 280 K, which seems to indicate the charge transport in this regime is dominated by acoustic phonon scattering (Biewald et al., 2019; Wright et al., 2016; Yi et al., 2016). Nevertheless, the influence of ion migration cannot be fully excluded in this regime (Yi et al., 2016). At lower temperatures, the mobility becomes almost independent on temperature, with *γ* being 0.04. The transition of the *μ-T* relationship at *T* = 200 K is suspected to be caused by the phase transition of (PEA)_2_SnI_4_ films, as discussed in the following. The weak temperature dependence of mobility was frequently reported in polycrystalline FETs, which was accounted by the combined effect of charge-carrier trapping at grain boundaries and phonon scattering in the grains (Katoh, 1994). This explanation may be applicable to the (PEA)_2_SnI_4_ films because of their polycrystalline nature but remains to be further investigated.

By comparison, the 5 mol % SnI_4_-doped devices present larger hysteresis (Figure 3H), which is probably due to the extra I^−^ brought by the SnI_4_ adding, as mentioned earlier. Interestingly, the mobility of the doped device shows a larger *γ* value of 3.23 in the range of 200–280 K. This large *γ* value is comparable to the ones observed in MAPbI_3_ FETs reported by Sirringhaus et al., which was believed to be accounted by ion migration (Senanayak et al., 2017). Thus, the larger *γ* in this regime suggests the more severe ion migration of the doped films, consistent with the more significant hysteresis shown earlier. In addition, an abrupt step change of mobility was seen at *T* = 200 K, which was reported to reflect the phase change of perovskite by Duan et al. (Yi et al., 2016). However, direct evidence showing the phase change of (PEA)_2_SnI_4_ films remains for further investigations, which is beyond the scope of this work. At *T* < 200 K, the *μ* keeps increasing as the *T* lowers, with *γ* being 0.64. Ion migration effect is not likely to account for this mobility increase because it is effectively suppressed at such low temperatures (Senanayak et al., 2017); however, the dominant mechanisms governing charge transport in this regime are not fully clear yet.

### Doping of (PEA)_2_SnI_4_ films for TE devices

Although the investigation of OIHP-based TE devices is still in its infancy and lags much behind that of traditional inorganic and organic TE devices, their advantages of cost-effectiveness as well as inherently ultralow thermal conductivity (*κ*) and high Seebeck coefficient (*S*) make OIHPs promising TE materials (Filippetti et al., 2016; Haque et al., 2020b; Shukla et al., 2020). Two-dimensional OIHPs possess even lower *κ* than their 3D counterparts (Giri et al., 2020), partly because the large acoustic mismatch between the organic and inorganic layers in 2D OIHPs was found to suppress the acoustic phonon transport (Guo et al., 2018). These electronic and thermal properties of 2D OIHPs are beneficial for achieving a high TE figure of merit (*ZT*) = *S*^2^*σT*/*κ*, where *σ* is the electrical conductivity and *T* is the absolute temperature. However, similar to organic semiconductors, the inherent carrier concentration and electrical conductivity in 2D OIHPs is rather low, which not only poses challenges for the characterization of their TE performance but also hinders the achievement of large power factor (*PF*, *PF* = *S*^2^*σ*) values (Cohen et al., 2017; Haque et al., 2020b; Mitzi et al., 1995). Here, with the doping technique shown earlier, we are allowed to evaluate the TE performance of (PEA)_2_SnI_4_ films by enhancing their *σ* values.

**Figure 4:**
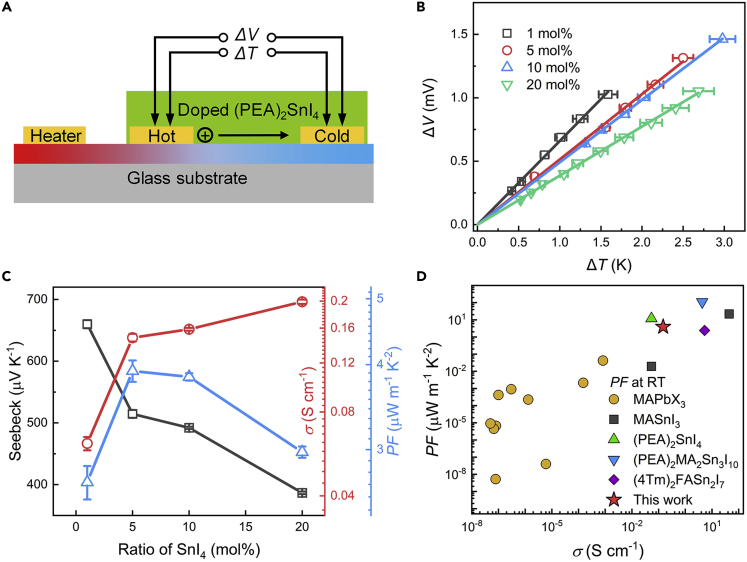
Thermoelectrical characterizations of doped (PEA)_2_SnI_4_ films (A) The schematic diagram of the thermoelectric measurements. (B) The dependence of thermal voltages on temperature differences for SnI_4_-doped (PEA)_2_SnI_4_ films. (C) Seebeck coefficients, electrical conductivities, and power factors of (PEA)_2_SnI_4_ films with different doping ratios. (D) A summary of the *PF* values versus corresponding electrical conductivities of 3D and 2D OIHP semiconductors at room temperature (X denotes the halide ions).

Figure 4A shows the schematic diagram of our setup for TE characterizations, which can provide information about *S* by measurements of temperature differences (Δ*T*) and the corresponding thermal voltages (Δ*V*) of the samples (see more information in the Method Details) (Wei et al., 2021). As shown in Figure 4B, Δ*V* becomes smaller with the increasing doping ratio at the same Δ*T*, indicating lower *S* (= Δ*V*/Δ*T*) at a higher doping ratio. Figure 4C shows the variation of TE parameters of (PEA)_2_SnI_4_ films with the doping ratio ranging from 1 mol % to 20 mol %. The average *S* extracted from Figure 4B reduced from 660 μV K^−1^ to 387 μV K^−1^, and the *σ* measured by the four-point probe method increased from 0.06 S cm^−1^ to 0.20 S cm^−1^. The TE characterizations of pristine devices were not conducted due to their low electrical conductivities. The highest *PF* value we have achieved is about 3.92 μW m^−1^ K^−2^ at the doping ratio of 5 mol %. A summary of previously reported *PF* values as a function of electrical conductivities for 3D and 2D OIHP semiconductors at room temperature (RT), including MAPbX_3_ (X denotes the halide ions) (Haque et al., 2019; Long et al., 2019; Mettan et al., 2015; Tang et al., 2020; Wu et al., 2018; Xie et al., 2021; Xiong et al., 2019; Ye et al., 2017), MASnI_3_ (Hao et al., 2014; Haque et al., 2020a), (PEA)_2_MA_2_Sn_3_I_10_ (Yang et al., 2021), and (4Tm)_2_FASn_2_I_7_ (Hsu et al., 2021), is shown in Figure 4D. It is seen that the *PF* value we have achieved in (PEA)_2_SnI_4_ by SnI_4_ doping is among the highest ones (Hsu et al., 2021; Yang et al., 2021). It is also notable that Sn-based OIHPs, which generally have higher *σ* than Pb-based OIHPs, exhibit higher *PF* values, suggesting that Sn-based OIHPs are very attractive for high-performance TE devices.

### Conclusion

In conclusion, we have reported an effective doping technique by utilizing SnI_4_ as a dopant for p-doping (PEA)_2_SnI_4_. The doping effect of Sn^4+^ was confirmed, and its influence on the electrical properties of (PEA)_2_SnI_4_ was revealed by electrical and spectroscopic characterizations. In addition to the doping effect, the incorporation of SnI_4_ was found to improve the film morphology of (PEA)_2_SnI_4_ films. By adopting the doping technique, we were able to enhance the device performance of (PEA)_2_SnI_4_ FETs dramatically, with mobility increased from 0.25 cm^2^ V^−1^ s^−1^ for pristine devices to 0.68 cm^2^ V^−1^ s^−1^ for 5 mol % SnI_4_-doped devices. Such performance enhancement can be attributed to the reduced contact resistance and charge trapping/scattering in the doped devices. Furthermore, we used the doping technique for improving the TE performance of (PEA)_2_SnI_4_, which shows a maximum of 3.92 μW m^−1^ K^−2^ at a doping ratio of 5 mol %. Such a high *PF* value demonstrates the great potential of 2D Sn-based perovskite for TE applications. Overall, our work provides a viable doping technique for (PEA)_2_SnI_4_ and, more importantly, shows their promising applications in enhancing the performance of devices.

### Limitations of the study

In this work, we have doped the Sn-based 2D perovskite (PEA)_2_SnI_4_ using SnI_4_ as a dopant. This strategy leads to significantly improved electrical conductivity and increased crystallite size. The FET and TE devices exhibited much improved mobilities and power factors after doping with SnI_4_, respectively. However, the charge scattering mechanisms in FETs at low temperatures are still unclear and require further investigations.

## STAR★Methods

### Key resources table

**Table:** 

REAGENT or RESOURCE	SOURCE	IDENTIFIER
**Chemicals, peptides, and recombinant proteins**		

Phenethylammonium Iodide, ≥99.5%	Xi’an Polymer	Cat#PLT501391I
Tin(II) iodide, AnhydroBeads™, −10 mesh, 99.99% trace metals basis	Aldrich	Cat#409308
*N,N*-Dimethylformamide, anhydrous, 99.8%	Aldrich	Cat#227056
1-Methyl-2-pyrrolidinone, anhydrous, 99.5%, packaged under Argon in resealable ChemSealTM bottles	Alfa Aesar	Cat#043741
Tin(IV) iodide ,95%	Aladdin	Cat#T195042

**Other**		

Keithley 4200 semiconductor analyzer	Tektronix Technologies	https://www.tek.com/en/keithley-4200a-scs-parameter-analyzer
Axis Supra spectrometer	Kratos	https://www.kratos.com/products/axis-supra-xps-surface-analysis-instrument
MIRA3 SEM	TESCAN	http://www.tescan-china.com/zh-cn/technology/special-solutions/mira3-amu
Park XE-7 AFM	Park Systems	https://parksystems.com/products/small-sample-afm/park-xe7/overview
D/max 2550 XRD	Rigaku	https://www.directindustry-china.cn/prod/rigaku/product-31512-886693.html
UV-3600 PLUS	SHIMADZU	https://shimadzu.com.au/uv-3600-plus
B2912A Precision Sources	Keysight	https://www.keysight.com/us/en/product/B2912A/precision-source-measure-unit-2-ch-10fa-210v-3a-dc-10-5a-pulse.html?rd=1
ST-100 cryostat	Janis	https://www.lakeshore.com/products/product-detail/janis/st-100-optical-cryostat

### Resource availability

#### Lead contact

Further information and requests for resources and reagents should be directed to and will be fulfilled by the lead contact, Yuanyuan Hu (yhu@hnu.edu.cn).

#### Materials availability

This study did not generate new unique reagents.

### Method details

#### Preparation of perovskite films

Sn^4+^ was introduced by mixing PEAI, SnI_2_ and SnI_4_ with mol ratio of 2:1−*x*:*x* (0 < *x* < 1) in a mixture of DMF and NMP with volume ratio of 3:1, where *x* represents the doping ratio of SnI_4_. 0.1 M (PEAI)_2_(SnI_2_)_1-*x*_(SnI_4_)_*x*_ precursor solutions were formed by heating at 60°C for 4 h in an Ar-filled glove box. Then, the precursor solutions were stored for 1 h to cool down to room temperature naturally and filtered through 0.45 μm polytetrafluoroethylene (PTFE) filters. Glass or Si^++^/SiO_2_ substrates were cleaned sequentially by deionized water, acetone and isopropanol, and blown dry by nitrogen gas. The substrates were treated with UV/ozone for 30 min before use. Perovskite films were prepared by spin-coating the precursor solution on the substrates at 4000 rpm for 30 s and annealing at 100°C for 10 min in an Ar-filled glove box.

#### Characterization of perovskite films

For electrical conductivity measurements, the perovskite films were deposited on SiO_2_ substrates with predefined electrodes (Cr/Au, 2 nm/30 nm). The conductivity of the prepared devices was measured using a probe station in an Ar glove box by four-point probe method through a Keithley 4200 semiconductor analyzer. XPS and UPS measurements were carried out by a Kratos Axis Supra spectrometer under a high vacuum. The morphologies of the films were investigated by SEM (MIRA3, TESCAN). The thicknesses of the films were measured by atomic force microscopy (AFM) (Park XE-7). The XRD patterns were recorded by D/max 2550 (Rigaku) under Cu Kα (λ = 1.5406 Å) irradiation. The steady PL spectra were recorded by a Thermo Scientific Lumina. The time-resolved PL measurements were performed using a confocal microscope (WITec, alpha-300) as the collect device, and the emission signal was reflected into a streak camera (C10910, Hamamatsu) by Ag mirrors. The laser beam (405 nm) was focused on the sample with a spot diameter of ≈3 μm from the top by an objective lens (50×, Zeiss, 0.75 NA), while PL emission was collected by the same objective lens. The ultraviolet-visible-near-infrared (UV-vis-NIR) absorption spectra of solution and film samples were measured with UV-3600 PLUS (SHIMADZU).

#### Fabrication and characterization of FETs

This study did not generate new unique reagents. Si^++^/SiO_2_ substrates with bottom-contact electrodes (Cr/Au: 2 nm/30 nm) defined by photolithography were cleaned sequentially by deionized water, acetone and isopropanol, and blown dry by nitrogen gas. The substrates were treated with UV/ozone for 30 min before use. The BGBC FETs were fabricated by spin-coating perovskite precursors on the SiO_2_ substrates at 4000 rpm for 30 s and annealing at 100°C for 10 min in an Ar-filled glove box. The transistor electrical characteristics at RT (about 25 °C) and in the dark were collected immediately after preparation using a probe station in an Ar-filled glove box through a Keysight B2912A Precision Sources in DC mode. For the temperature-dependent measurement, the device was loaded into a Janis ST-100 cryostat in an Ar-filled glove box, and the electrical measurements were taken in vacuum with pressure lower than 1 × 10^−5^ mbar. All measurement processes were carried out under dark conditions. The saturation FET mobility was calculated from both forward and backward curves using

$$\mu_{\text{sat}}=\frac{2L}{WC_{\text{i}}}{\left(\frac{\partial \sqrt{\left|I_{\mathrm{DS}}\right|}}{\partial \sqrt{\left|V_{\mathrm{GS}}\right|}}\right)}^{2}$$

where *L*, *W* and *C*_i_ are the channel length, width, and dielectric area capacitance, respectively.

#### Measurement of Seebeck coefficients

Thermoelectric devices were fabricated by the same spin-coating process on the cleaned glass substrates. To measure the Seebeck coefficients of doped (PEA)_2_SnI_4_ films, a homemade thermoelectric measurement system was used. The devices containing one heater, two thermometers which also act as electrical contacts were fabricated by photolithographic patterning of metal bilayers of Cr (10 nm) and Au (15 nm) on glass substrates. To obtain Seebeck coefficient *S* = Δ*V*/Δ*T*, the temperature gradient between the two electrodes was estimated by converting the resistance of electrodes into temperature using the temperature-coefficient-of resistance (TCR), and the built-in thermal voltage was measured using Keithley Nanovoltmeter model 2182A. All Seebeck coefficients were measured at RT in a high vacuum (<10^−5^ mbar) using Janis ST-100 in the dark.

## Data Availability

Any additional information required to reanalyze the data reported in this paper is available from the lead contact upon request.
