# Supplementary material for: Doping of Sn-based two-dimensional perovskite semiconductor for high-performance field-effect transistors and thermoelectric devices
Source: iScience. 2022 Mar 17;25(4):104109. doi: 10.1016/j.isci.2022.104109 (PMC8983347; doi:10.1016/j.isci.2022.104109)
Supplement: Document S1. Figures S1–S8 [file mmc1.pdf]

iScience, Volume *25*

Supplemental information

Doping of Sn-based two-dimensional perovskite semiconductor for high-performance field-effect transistors and thermoelectric devices

Yu Liu, Ping-An Chen, Xincan Qiu, Jing Guo, Jiangnan Xia, Huan Wei, Haihong Xie, Shijin Hou, Mai He, Xiao Wang, Zebing Zeng, Lang Jiang, Lei Liao, and Yuanyuan Hu

Supplemental Figures

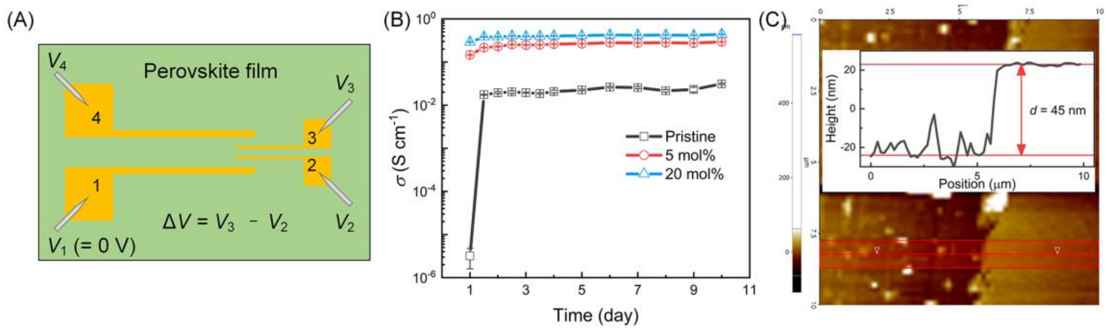

Figure S1. Conductivity measurement of $(PEA)_2SnI_4$ doped by SnI_4, Related to Figure 1.

(A) Four-point probe method measurement diagram.

(B) Time-dependent electrical conductivities of pristine $(PEA)_2SnI_4$ films and films doped with 5 mol% and 20 mol% SnI_4.

(C) The thicknesses of films (~45 nm) measured by atomic force microscopy (AFM).

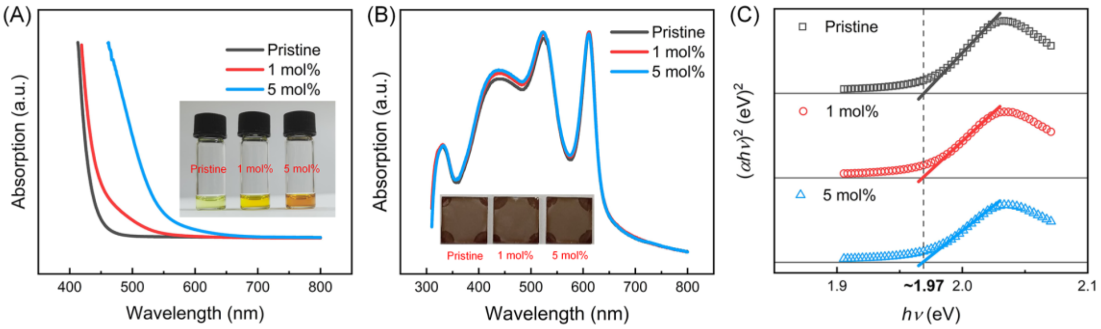

Figure S2. Spectral characterizations of $(PEA)_2SnI_4$ doped by SnI_4, Related to Figure 2.

(A) Absorption spectra of pristine, 1 mol%, and 5 mol% SnI_4-doped $(PEA)_2SnI_4$ precursors. Inset: photos of perovskite precursors.

(B) The absorption spectra of pristine, 1 mol% and 5 mol% SnI_4-doped $(PEA)_2SnI_4$ films. Inset: photos of perovskite films spin-coated on glass substrates.

(C) Tauc plots of pristine, 5 mol% and 20 mol% SnI_4-doped $(PEA)_2SnI_4$ films for optical bandgap extraction.

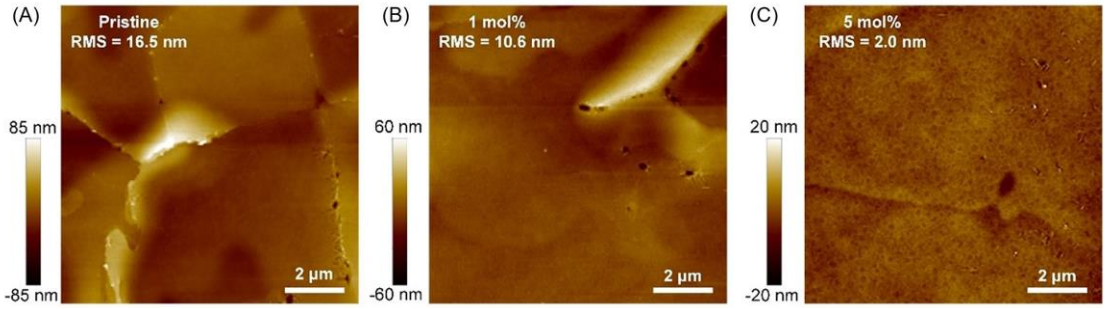

Figure S3. Morphology characterizations of $(PEA)_2SnI_4$ doped by SnI_4, Related to Figure 2.

AFM images of (A) pristine, (B) 1 mol% and (C) 5 mol% SnI_4-doped $(PEA)_2SnI_4$ films with roughness of 16.5 nm, 10.6 nm and 2.0 nm, respectively. Scale bar: 2 μm.

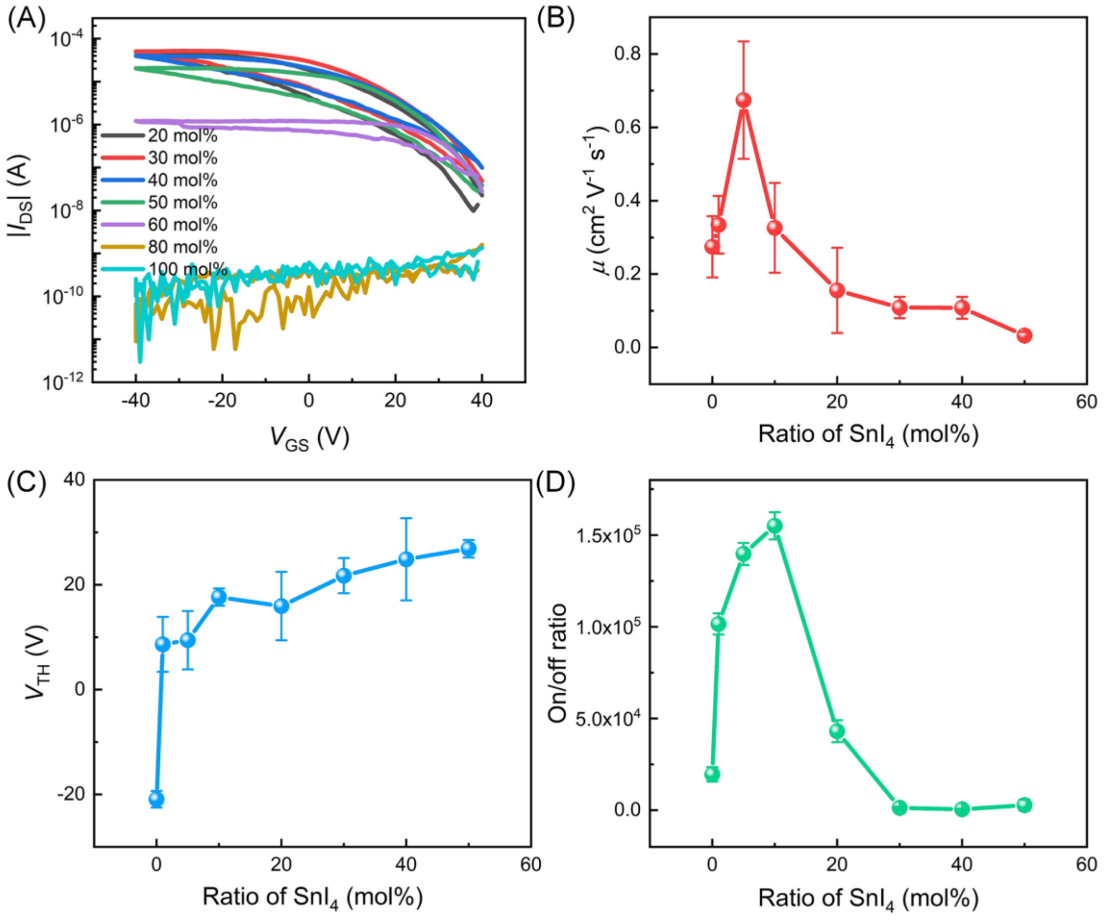

Figure S4. FETs performance of $(PEA)_2SnI_4$ doped by high SnI_4 ratios, Related to Figure 3.

(A) Transfer characteristics of FETs with high SnI_4 doping ratio (≥ 20 mol%).

(B) Static (B) mobility, (C) V_{TH} and (D) on/off ratio as a function of SnI_4 doping ratio.

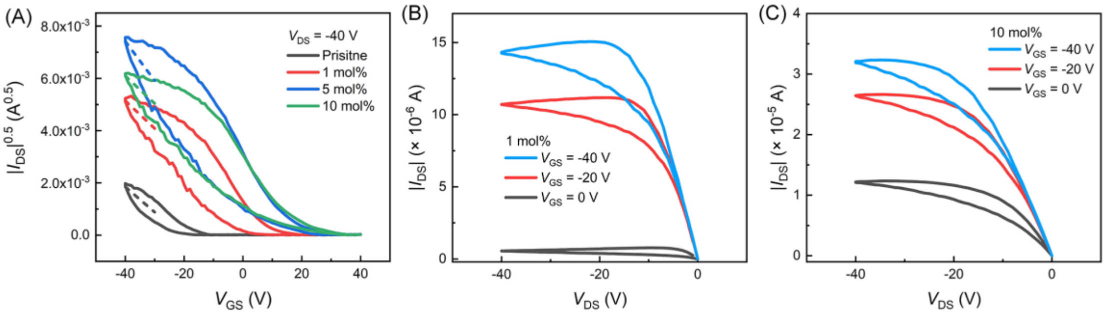

Figure S5. FETs measurement of $(PEA)_2SnI_4$ doped by SnI_4, Related to Figure 3.

(A) Transfer plots (the square root of current) with different doping ratios. The dash lines represent the linear fitting of forward and backward curves simultaneously (V_{GS}: -40 V ~ -30 V) for mobility calculation.

Output characteristics of (B) 1 mol% and (C) 10 mol% SnI_4-doped FETs.

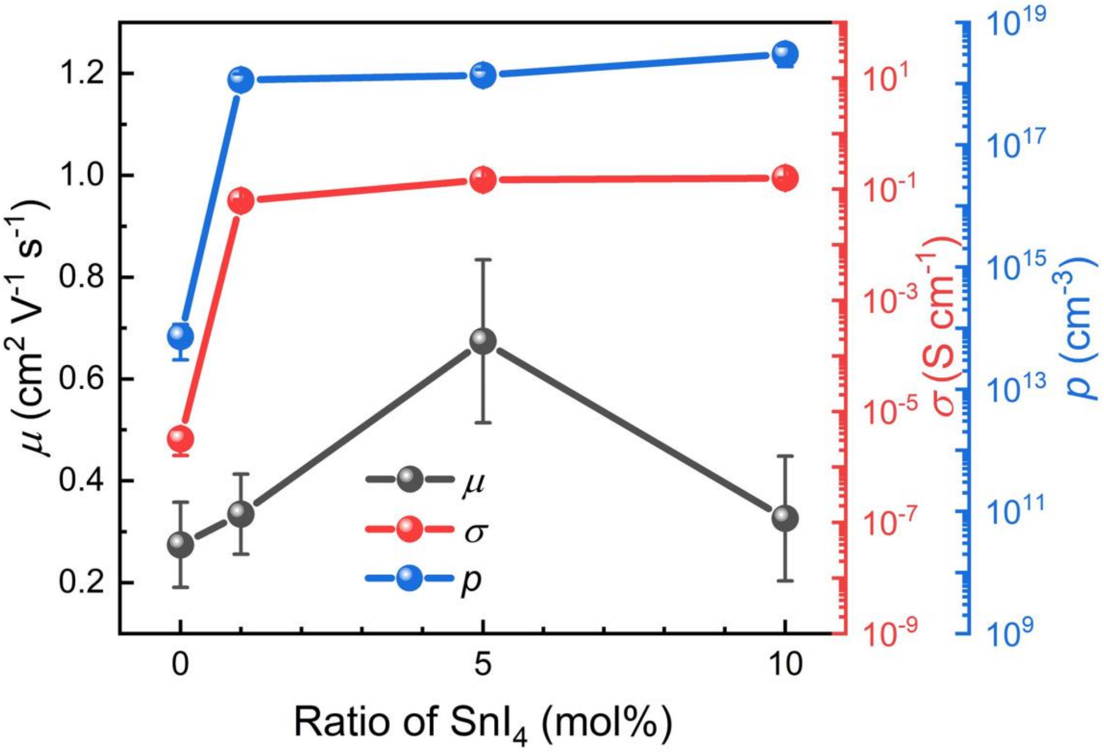

Figure S6. Field-effect mobility μ, electrical conductivity σ and carrier concentration p of pristine, 1 mol%, 5 mol% and 10 mol% SnI_4-doped $(PEA)_2SnI_4$ films, Related to Figure 3.

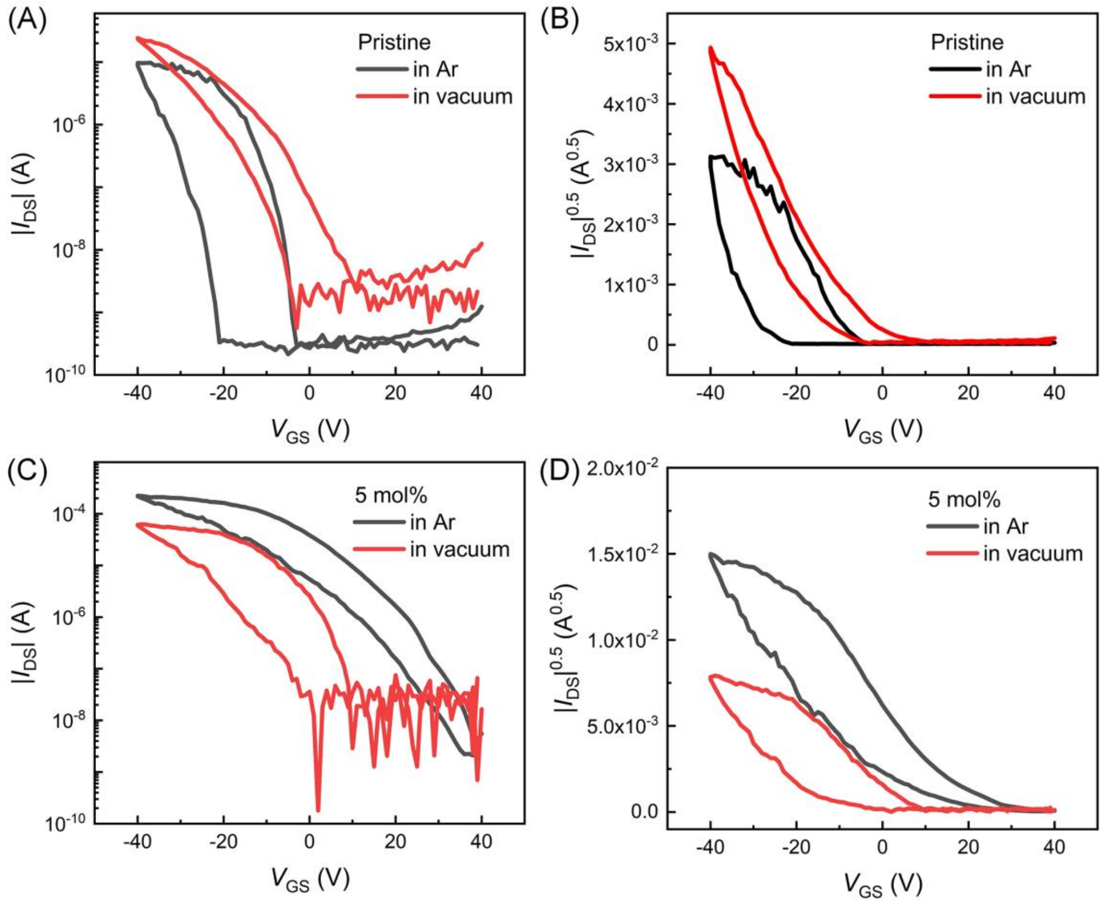

Figure S7. The change of transfer curves after vacuuming, Related to Figure 3.

The transfer characteristics of (A)(B) pristine and (C)(D) 5 mol% SnI_4-doped FETs before and after vacuuming.

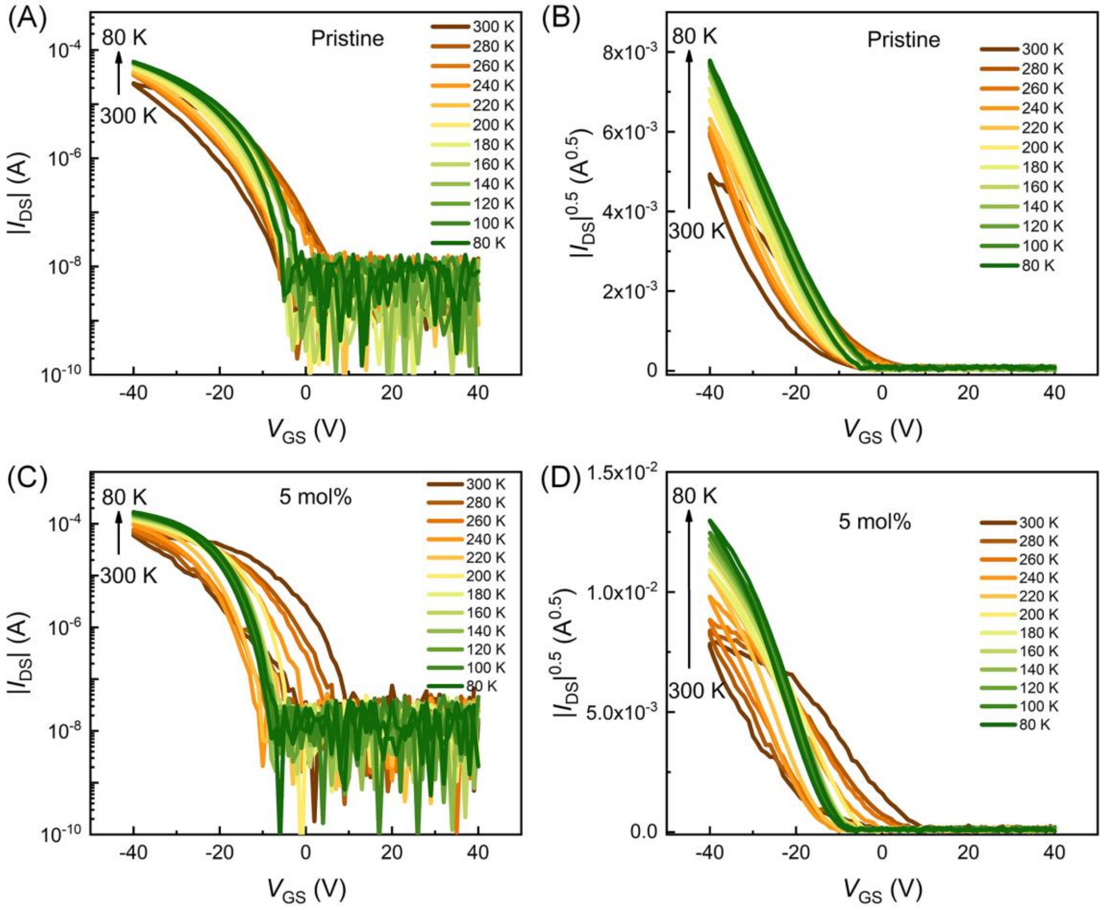

Figure S8. Temperature-dependent FETs transfer characteristics with decreased temperature from 300 K to 80 K, Related to Figure 3.

(A)(B) pristine FETs and (C)(D) 5 mol% SnI_4-doped FETs.
